# Template Bias in AlphaFold 3 Impedes Analysis of Point Mutations

**DOI:** 10.34133/csbj.0240

**Published:** 2026-11-12

**Authors:** Juliane Lokau, Isabel Ramón Roth, Jana I. Führing, Johannes N. Greve, Stefan Düsterhöft, Christoph Garbers

**Affiliations:** ^1^ Institute of Clinical Biochemistry, Hannover Medical School, 30625 Hannover, Germany.; ^2^ Institute for Biophysical Chemistry, Hannover Medical School, Fritz-Hartmann-Centre for Medical Research, Hannover, Germany.; ^3^ Institute of Molecular Pharmacology, Medical Faculty, RWTH Aachen University, Aachen, Germany.; ^4^ HMU Health and Medical University, Campus Düsseldorf/Krefeld, Düsseldorf, Germany.

## Abstract

Cytokine receptors are important therapeutic targets in anti-inflammatory therapy. Point mutations in the interleukin-6 receptor and the interleukin-11 receptor are associated with inflammatory disorders and developmental defects of the skull, respectively. In silico methods to determine whether a novel point mutation found in a patient is pathogenic and thus the likely cause for the patients’ disease are therefore of high diagnostic interest. There is an ongoing discussion about whether AlphaFold 3 (AF3) is a suitable tool to understand the influence of point mutations on the structure and hence function of a protein. In this study, by analyzing all interleukin-6 receptor and interleukin-11 receptor variants that are known to be disease associated, we find that the structural models generated by AF3 are more similar to solved crystal structures of the wild-type (WT) cytokine receptors than the corresponding WT models. We show that this over-accordance is caused by template bias of AF3, whereby the best-fitting template is not used for the WT models, but is used when a single amino acid is altered in the input sequence. Our results suggest that AF3 should be used with extreme caution when comparing WT proteins and single-point mutations to assess structural changes underlying disease association.

## Introduction

Cytokines are small secreted proteins that bind to membrane-bound receptors on their target cells in order to activate intracellular signaling cascades. Cytokines of the interleukin-6 (IL-6) family have pleiotropic functions in the human body, ranging from developmental processes over tissue homeostasis and immune cell regulation to deleterious effects manifesting as inflammatory diseases. Consequently, inhibition of especially IL-6 is used in clinical practice to treat patients with inflammatory diseases [1–3]. Antibodies blocking the biological activity of interleukin-11 (IL-11) are currently in clinical trials for fibrosis treatment [4].

There is ample evidence that mutations within the genes encoding the interleukin-6 receptor (IL-6R) and the interleukin-11 receptor (IL-11R) are causative for human diseases. Point mutations of the IL-6R have been described in a patient with immunodeficiency, atopy, and abnormal inflammatory responses [5]. Craniosynostosis, a condition in which the sutures that connect the head bones close prematurely and cause a malformed skull, is associated with point mutations of the IL-11R [6–8]. We have shown previously that these mutations within *IL11RA* cause a loss-of-function of the IL-11R protein [9–11]. The underlying molecular reason appears to be a folding defect of the IL-11R, which results in impaired receptor maturation and a subsequent lack of transport to the cell surface.

The development of the artificial intelligence program AlphaFold (AF) and its derivatives has revolutionized structural biology [12–15]. Consequently, the usage of AF for the prediction of the structure and function of a protein is increasing [16]. Since predicting the influence of point mutations on the structure and function of the protein in silico is of high interest, several studies have tried to evaluate whether AF can be leveraged for such purposes [17–22]. However, the results of these approaches are rather mixed, and it is still unclear whether AlphaFold 3 (AF3) represents a valid approach to analyze point mutations in therapeutically relevant proteins. Therefore, it is particularly important to clearly highlight the potential pitfalls and limitations of such predictions.

In this study, we generated and analyzed structural models of all known disease-associated IL-6R and IL-11R variants by AF3. We find that structural models of single-point mutations differ globally from the wild type (WT). However, these differences do not reflect AF3 detecting the impact of the mutation itself, but rather represent an over-accordance toward experimentally solved crystal structures of the cytokine receptors, driven by a template selection bias in AF3.

## Materials and Methods

### Selection of cytokine receptor variants

For this study, we included all craniosynostosis-associated IL-11R variants with single-point mutations that we are aware of (most of them are listed in [6]). For the nonpathogenic IL-11R variants, we screened the Genome Aggregation Database (gnomAD v4.1.0 [23]) and selected only variants for which at least one homozygous individual is annotated. For 2 of these variants, we have previously shown that they do not prevent the biological function of the IL-11R [24]. For the IL-6R, we included 2 variants known as pathogenic [5] as well as 3 nonpathogenic variants identified by us (manuscript under consideration elsewhere).

### Generation of cytokine receptor sequences with random mutations

In order to generate IL-6R and IL-11R variants with random mutations, we used the Sequence Manipulation Suite ([25], available at https://www.bioinformatics.org/sms2/mutate_protein.html). We ran the program 10 times separately per receptor to generate 10 different variants each with a single mutation within the amino-acid sequence.

### Generation of IL-6R and IL-11R structural models with AF3

The primary sequences of the human IL-6R (UniProt ID: P08887) and the human IL-11R (UniProt ID: Q14626) were obtained from the UniProt database. Like in our previous study [26], we removed the signal peptide, the stalk region, the transmembrane, and the intracellular region of both receptors and generated the structural models only with the ectodomains (domains D1, D2, and D3 [27]) of the 2 cytokine receptors. This was necessary because the crystal structures of the IL-6R (PDB: 1n26, [28]) and the IL-11R (PDB: 6o4p, [29]) consist only of the ectodomains and do not include the signal peptide, the stalk region, the transmembrane, and the intracellular region.

We used AF3 ([12], available at https://alphafoldserver.com/) to predict the structures of IL-6R-WT, IL-11R-WT, and the different variants with point mutations using the default settings including usage of Protein Data Bank (PDB) templates with the default cutoff date (2021 Sep 30). We analyzed either 25 models per receptor variant, which were derived from 5 independent seeds, or 5 models per receptor variant derived from 1 seed, as described in the respective figure legends. For the experiments using a defined set of templates, we performed the computations on the high-performance cluster of the Hannover Medical School Information Technology Centre. Custom templates were prepared by removing all protein chains except the template chain from the respective PDB files, as required for the use of custom templates in AF3. When custom templates are supplied, AF3 does not automatically generate multiple sequence alignments (MSAs) and therefore requires MSAs to be provided explicitly by the user. To obtain these MSAs, default AF3 predictions were performed using the respective query sequences. The MSAs generated during these default runs were extracted from the JavaScript Object Notation output files using custom Python scripts and subsequently used for predictions with the defined set of templates.

### Analysis of cytokine receptor models

We used ChimeraX (version 1.7.1) to analyze protein structures [30]. As described in detail previously [26], we performed structural comparisons using the Matchmaker algorithm. For each comparison, we determined the root-mean-square deviation (RMSD) across all aligned Cα atom pairs, the number of Cα atom pairs retained after iterative pruning, and the RMSD of the retained atom pairs. Matchmaker pruning was performed using the default iterative pruning settings with a cutoff distance of 2.0 Å. Importantly, the RMSD across all aligned Cα atom pairs was calculated independently of the pruning step and therefore allowed direct comparison between the individual structural models.

We compared the structural models with solved protein structures deposited in the PDB; the accession numbers of the used structures are mentioned in the text, the figures, or within the respective figure legends. Importantly, the number of pruned atom pairs and the corresponding RMSD of these atom pairs can differ between the WT receptors and their variants. Determining of the total RMSD, which comprises all Cα and is therefore constant over all structural models, ensures comparability between the individual analyses. For the analysis of the 3 individual IL-11R domains, we split the IL-11R ectodomain into 3 parts according to their domains: Domain D1 contained amino-acid residues 21 to 112, domain D2 contained amino-acid residues 113 to 217, and domain D3 contained amino-acid residues 218 to 316. The individual domains were then compared to the IL-11R crystal structure as described above. For the quantitative analysis of the AF3 models, predicted local distance difference test (pLDDT), predicted template modeling (pTM), and predicted aligned error (PAE) scores were extracted from the respective AF3 output files. Per-residue pLDDT values and PAE matrices were averaged across all predictions. Changes in predicted structural uncertainty were assessed as ΔPAE between the respective variant and WT. Data processing was performed using NumPy (version 1.24.3) and Pandas (version 2.3.3), and graphical representations were generated using Matplotlib (version 3.9.2).

### Analysis of point mutations using AlphaMissense

The analysis of IL-11R and IL-6R point mutations was performed using AlphaMissense [31] via https://alphamissense.hegelab.org/. Here, data for both IL-11R and IL-6R were retrieved using the standard values without any customized values for the high-pass and low-pass cutoffs for pathogenicity scores. The classification of the variants as either “likely benign”, “likely pathogenic”, or “ambiguous” was then visualized using GraphPad Prism 10 (GraphPad Software, Boston, MA, USA).

### Statistical analyses

Statistical analyses were carried out using GraphPad Prism 10. Statistical differences were assessed using one-way analysis of variance (ANOVA) combined with Dunnett’s multiple comparisons test. Further details are given in the figure legends.

## Results and Discussion

### Structural models of IL-11R variants with point mutations are more similar to the IL-11R crystal structure than the model of IL-11R-WT

Mutations within the *IL11RA* gene, which encodes the IL-11R, are associated with craniosynostosis [6]. In order to investigate whether structural models generated by AF3 could be reliably used to determine the influence of such point mutations on the overall structure of the IL-11R, we generated structural models for 15 known craniosynostosis-associated IL-11R variants and IL-11R-WT (Fig. 1A). Importantly, we have previously shown for some of these variants that they cause retention within the endoplasmic reticulum when expressed in cells, most likely due to misfolding of the IL-11R [9–11]. The crystal structure of the IL-11R has recently been published (PDB: 6o4p, [29]), which allows us to directly compare the structural models generated by AF3 with the experimentally solved 3-dimensional structure of the protein (Fig. S1A). For this, we determined the RMSD for the whole structural model, the number of pruned atom pairs, and the RMSD of these pruned atom pairs. Higher total RMSD values and numbers of Cα atom pairs retained after pruning indicate greater structural divergence between an AF3-generated model and the experimentally determined WT crystal structure. As we have previously shown that the pathologic IL-11R mutations compromise the function of the protein [9–11], we can reasonably assume that the overall structure differs from WT. Therefore, if the AF3 models of these variants showed higher RMSD values, this would indicate that AF3 could indeed be able to account for structural alterations caused by the point mutations (Fig. S1B). Surprisingly, the opposite was the case, as all IL-11R variants had highly significantly lower total RMSD values (Fig. 1B) and significantly higher numbers of pruned atom pairs than the IL-11R WT model when compared to the IL-11R crystal structure (Fig. 1C). Importantly, the RMSD values of these pruned atom pairs did not differ for most variants, indicating overall a good match of all modeled structures to the IL-11R crystal structure (Fig. 1D). Interestingly, most IL-11R variants showed highly similar PAE patterns, pLDDT profiles, and pTM scores and exhibited only small deviations from IL-11R-WT (Fig. S2A to C). This was largely independent of the position of the respective mutation. The exceptions were P221R and G110C, which affect residues located at domain boundaries. In particular, the PAE profiles of P221R and G110C showed reduced confidence in the relative positioning of the receptor domains, suggesting that interdomain interactions are less consistently predicted, probably due to their greater flexibility. This effect was most pronounced for P221R, which also showed lower pLDDT values and reduced, more variable pTM scores.

**Fig. 1.**
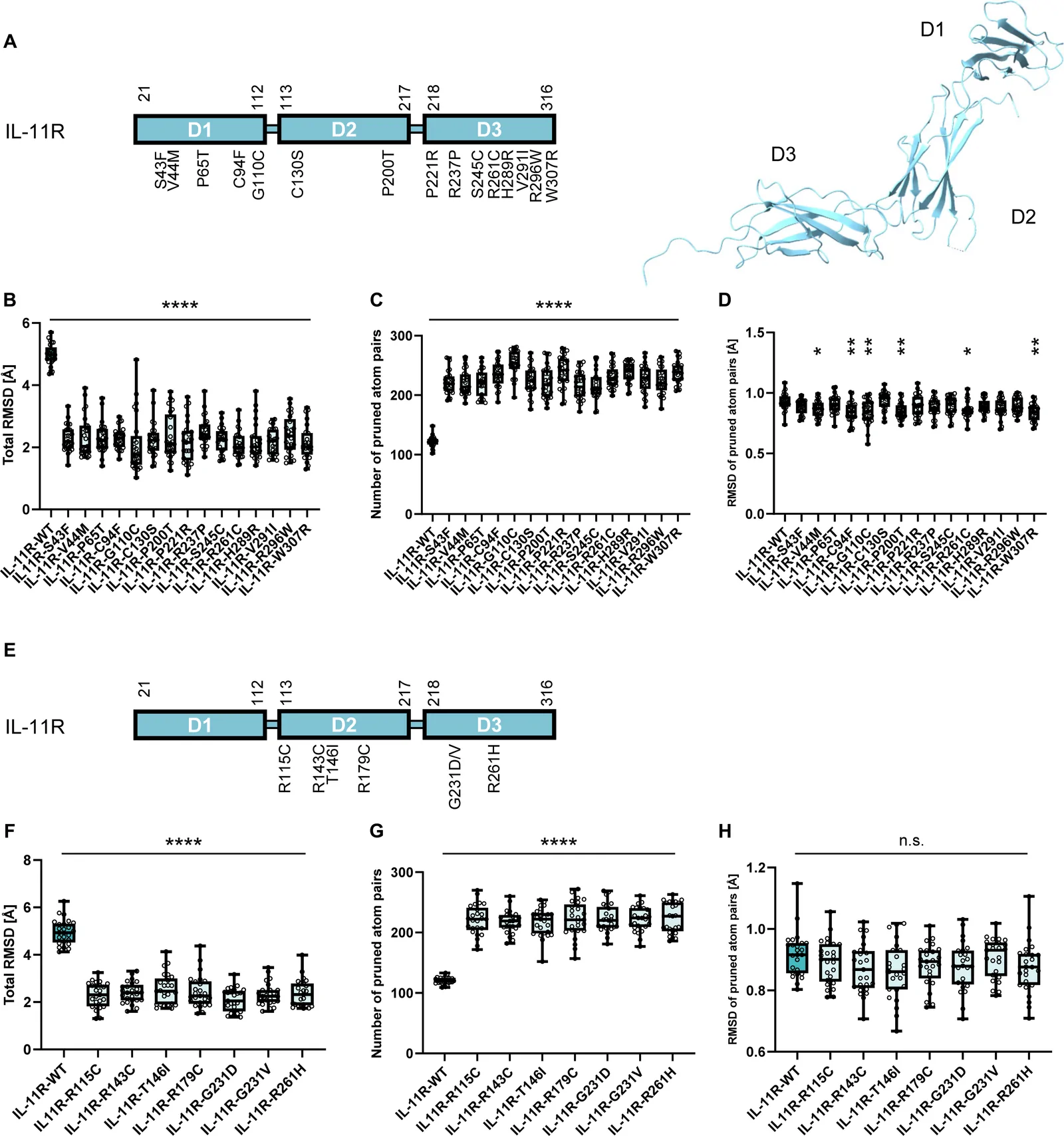
Structural models of IL-11R variants with point mutations fit better to the IL-11R crystal structure than the model of IL-11R-WT. (A) Schematic overview of the 3 extracellular domains (D1, D2, and D3) of the IL-11R. Numbers above the domains indicate the first and last residue according to [27]. The craniosynostosis-associated IL-11R variants are given below the IL-11R. The protein structure of the IL-11R is shown on the right, and the 3 domains are indicated (PDB: 6o4p). (B) Total root-mean-square deviation (RMSD) values (Å) of the structural models of IL-11R-WT and the different IL-11R variants when compared to the IL-11R crystal structure. The values of the 25 models (derived from 5 independent seeds) per variant are shown as a box-and-whiskers plot with all individual data points indicated. (C) Number of pruned atom pairs of the structural models of IL-11R-WT and the different IL-11R variants when compared to the IL-11R crystal structure. The values of the 25 models (derived from 5 independent seeds) per variant are shown as a box-and-whiskers plot with all individual data points indicated. (D) RMSD values (Å) of the pruned atom pairs shown in panel C. The values of the 25 models (derived from 5 independent seeds) per variant are shown as a box-and-whiskers plot with all individual data points indicated. (E) Schematic overview of the 3 extracellular domains (D1, D2, and D3) of the IL-11R. The IL-11R variants derived from gnomAD are given below the IL-11R. (F to H) Data for the gnomAD variants are shown as described in the legends to panels B to D. All variants were compared to the IL-11R-WT with a one-way ANOVA with Dunnett’s multiple comparisons test. Statistically significant differences are indicated as follows: *P < 0.05; **P < 0.01; ****P < 0.0001; n.s., no significant difference.

In order to find out whether this is a general phenomenon and also occurs with non-disease-associated variants, we next analyzed 7 IL-11R variants from the gnomAD database for which at least one homozygous individual is annotated (Fig. 1E and Fig. S1C). We have previously shown that some of these mutations do neither prevent expression of the IL-11R variants at the cell surface nor impair the biological functions of the IL-11R [24]. In line with our previous results, we observed significantly reduced overall RMSD values (Fig. 1F), significantly increased number of pruned atom pairs (Fig. 1G), and again no significant difference in RMSD values of these pruned atom pairs (Fig. 1H). Similarly, the different IL-11R variants showed highly similar PAE patterns and pLDDT profiles, with only minor deviations from IL-11R-WT (Fig. S3A and B). pTM scores were also comparable among the variants, although consistently slightly lower than for IL-11R-WT (Fig. S3C). These changes were again largely independent of the position of the respective mutation. We concluded that the observed behavior of AF3 is independent of whether the point mutation is deleterious to the receptor’s function or not.

### Higher similarity of the structural models of the IL-11R variants is mostly dependent on the D2 and D3 domain and independent of the localization of the point mutation

The IL-11R consists of 3 extracellular domains, which are termed D1, D2, and D3 [27], and our analyzed craniosynostosis-associated point mutations are distributed over all 3 domains (Fig. 1A). Therefore, we next investigated whether our observed effect is caused by the domain which contains the mutation or whether the higher similarity of the structural models is independent of the actual mutation. For this, we compared the 3 domains individually with the IL-11R crystal structure and determined the same 3 parameters as before. For the 5 mutations that are localized within the D1 domain, we noted a significantly reduced total RMSD for IL-11R-C94F and IL-11R-G110C compared to IL-11R-WT, while the other 3 variants did not differ significantly from IL-11R-WT (Fig. 2A). All the variants that did not contain a mutation within the D1 domain were also not different from IL-11R-WT (Fig. 2A). We noted a significantly increased number of pruned atom pairs for IL-11R-S245C, IL-11R-R261C, and IL-11R-V291I, all located in the D3 domain, while all other variants, including the 5 with a mutation within the D1 domain, did not differ from IL-11R-WT (Fig. 2B). Interestingly, the RMSD values of the pruned atom pairs were significantly different for all variants compared to IL-11R-WT, irrespective of whether the D1 domain contained a mutation or not (Fig. 2C). We concluded from this that those structural models with a mutation within the D1 domain do not differ from the models with a mutation in one of the other domains when only the D1 domain is compared to the crystal structure of IL-11R-WT.

**Fig. 2.**
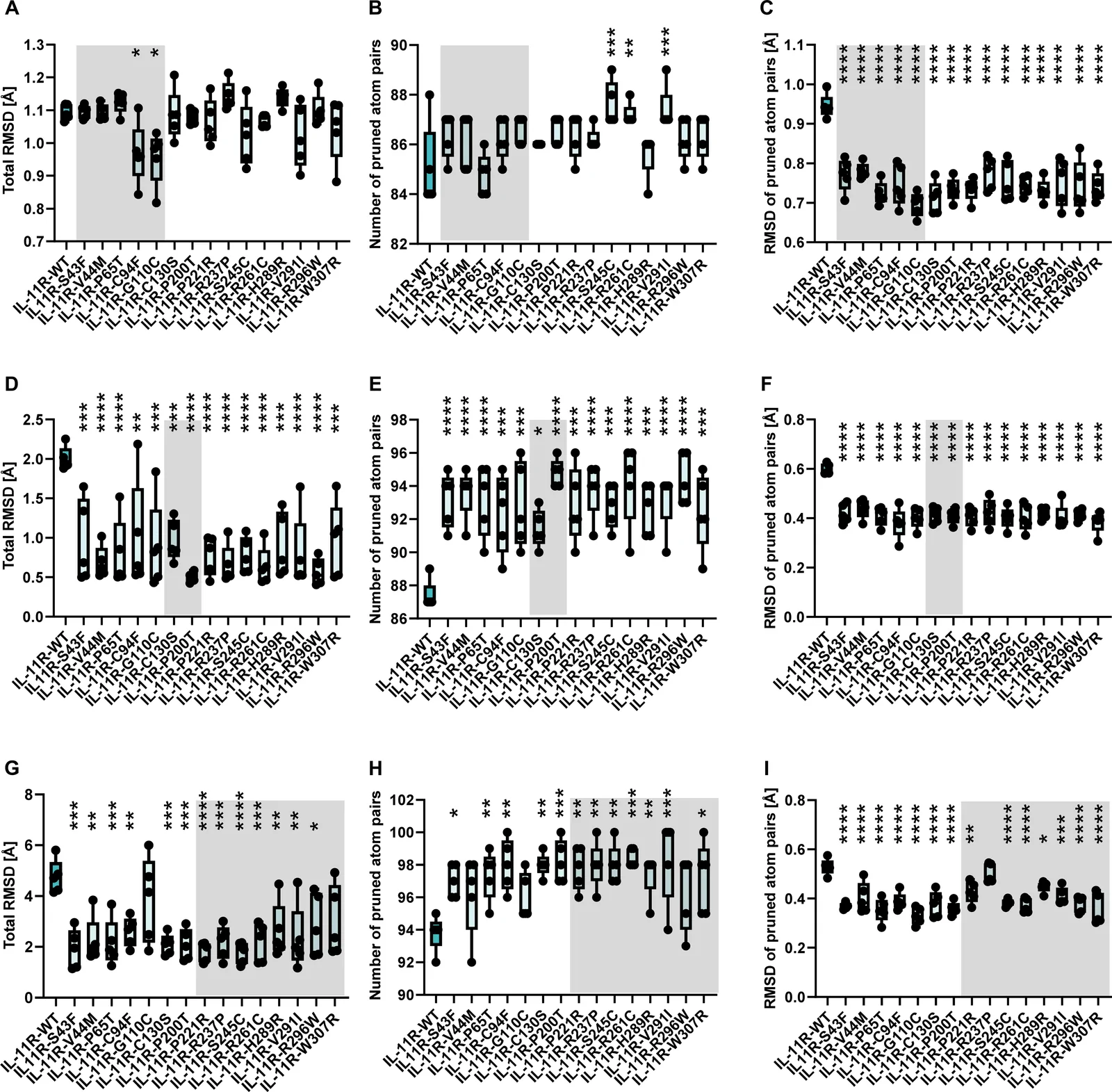
Higher similarity of the structural models of the IL-11R variants is mostly dependent on the D2 and D3 domains and independent of the localization of the point mutation. (A) Total RMSD values (Å) of the D1 domains of the structural models of IL-11R-WT and the different IL-11R variants when compared to the IL-11R crystal structure. The values of the 5 models per variant are shown as a box-and-whiskers plot with all individual data points indicated. (B) Number of pruned atom pairs of the D1 domains of the structural models of IL-11R-WT and the different IL-11R variants when compared to the IL-11R crystal structure. The values of the 5 models per variant are shown as a box-and-whiskers plot with all individual data points indicated. (C) RMSD values (Å) of the pruned atom pairs shown in panel B. The values of the 5 models per variant are shown as a box-and-whiskers plot with all individual data points indicated. (D to F) The analyses were performed as described for panels A to C, but the D2 domains of the structural models were analyzed. (G to I) The analyses were performed as described for the panels A to C, but the D3 domains of the structural models were analyzed. The gray background indicates that for these variants the mutation is located within the analyzed domain that is analyzed. All variants were compared to the IL-11R-WT with a one-way ANOVA with Dunnett’s multiple comparisons test. Statistically significant differences are indicated as follows: *P < 0.05; **P < 0.01; ***P < 0.001; ****P < 0.0001. All other variants showed no significant difference.

We then performed the same analysis for the D2 and D3 domains. For the D2 domain, all structural models were significantly different from the model of IL-11R-WT in terms of total RMSD (Fig. 2D), the number of the pruned atom pairs (Fig. 2E), and the RMSD of these atom pairs (Fig. 2F). Similarly, the vast majority of the D3 domains differed significantly from the IL-11R-WT model with regard to total RMSD (Fig. 2G), the number of pruned atom pairs (Fig. 2H), and their RMSD (Fig. 2I) independent from the location of the mutation.

We concluded from these analyses that the higher similarity of the structural models of the IL-11R variants with point mutations to the experimental IL-11R structure is mainly driven by the D2 and D3 domains, and less by the D1 domain, and that this is independent from the localizations of the actual point mutation. Intriguingly, while we did not observe a difference in the RMSD of the pruned atom pairs for the whole structural models (Fig. 1D), this was detectable for most of the individual domains.

### Structural models of IL-6R variants with point mutations are more similar to the IL-6R crystal structure than the model of IL-6R-WT

In order to determine whether this effect is restricted to the IL-11R, we next analyzed 2 known pathogenic point mutations (I279N and H280P, [5]) and 3 nonpathogenic variants of the IL-6R (Q154R, E182A, and S185R; Fig. 3A and Fig. S4). Just like with the IL-11R, a crystal structure also exists for the IL-6R (PDB: 1n26, [28]), allowing the direct comparison of the structural models of IL-6R-WT and the structural models of the different IL-6R variants with an experimentally derived 3-dimensional structure. Both pathogenic variants had significantly lower total RMSD values and significantly higher number of pruned atom pairs than the IL-6R-WT model (Fig. 3B and C). Interestingly, even the RMSD of the pruned atom pairs was lower compared to the IL-6R-WT model, indicating that only the aligned part of the variants matched the crystal structure better than the IL-6R-WT (Fig. 3D). We obtained the same results for the 3 nonpathogenic IL-6R mutations (Fig. 3E and F). In line with this, both the pathogenic (Fig. S5A to C) and nonpathogenic IL-6R variants (Fig. S5D to F) showed highly similar PAE and pLDDT profiles with only modest deviations from IL-6R-WT. The pTM scores were also highly comparable among the respective variants but were consistently slightly higher than for IL-6R-WT. Overall, no pronounced variant-specific alterations were observed, and the minor differences compared to IL-6R-WT did again not appear to depend on the position of the respective amino-acid substitution.

**Fig. 3.**
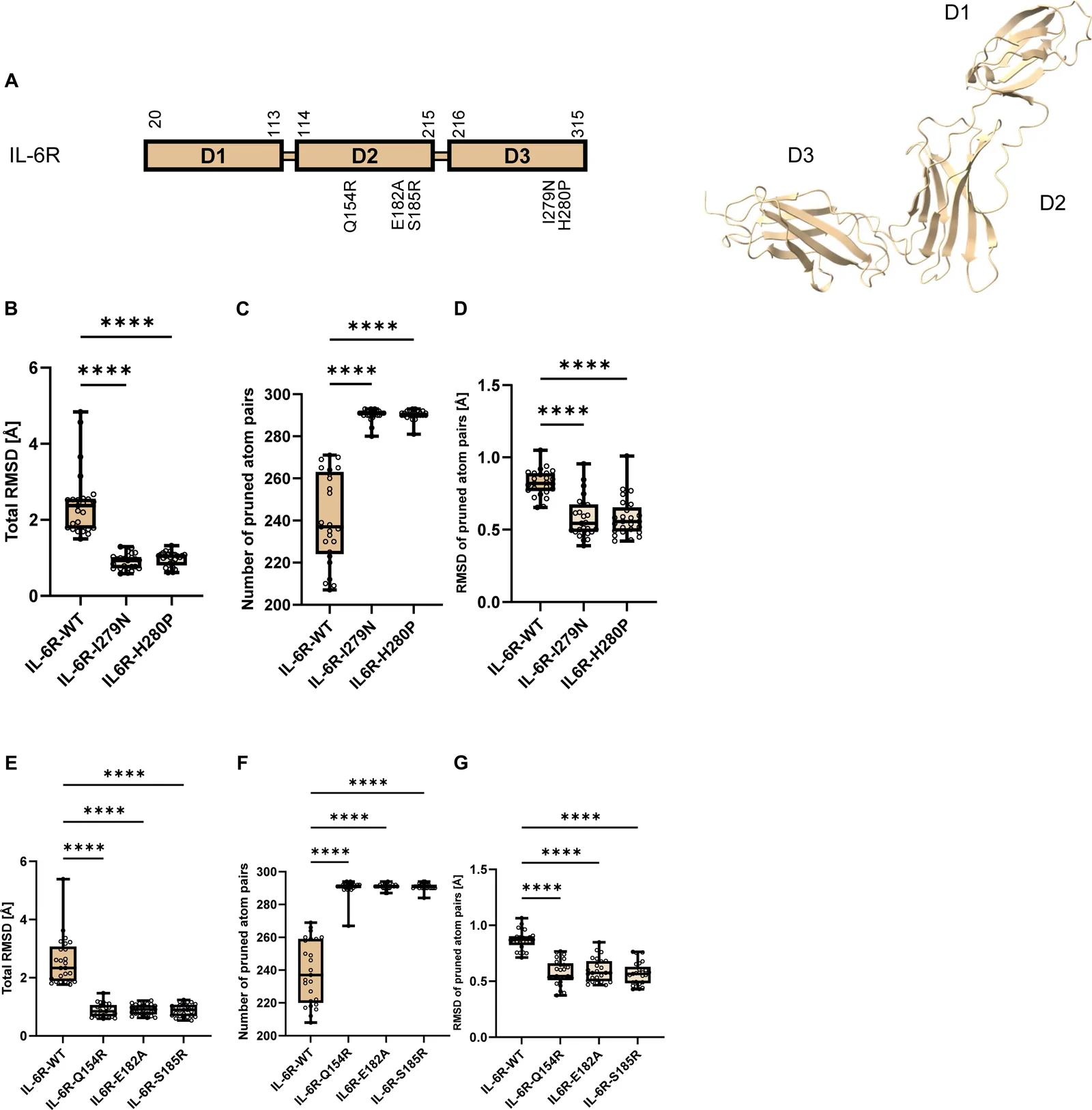
Structural models of IL-6R variants with point mutations fit better to the IL-6R crystal structure than the model of IL-6R-WT. (A) Schematic overview of the 3 extracellular domains (D1, D2, and D3) of the IL-6R. Numbers above the domains indicate the first and last residue according to [27]. The 2 disease-associated and the 3 nonpathogenic IL-6R variants are given below the IL-6R. The protein structure of the IL-6R is shown on the right, and the 3 domains are indicated (PDB: 1n26). (B) Total RMSD values (Å) of the structural models of IL-6R-WT and the 2 pathogenic IL-6R variants when compared to the IL-6R crystal structure. The values of the 25 models (derived from 5 independent seeds) per variant are shown as a box-and-whiskers plot with all individual data points indicated. (C) Number of pruned atom pairs of the structural models of IL-6R-WT and the 2 pathogenic IL-6R variants when compared to the IL-6R crystal structure. The values of the 25 models (derived from 5 independent seeds) per variant are shown as a box-and-whiskers plot with all individual data points indicated. (D) RMSD values (Å) of the pruned atom pairs shown in panel C. The values of the 25 models (derived from 5 independent seeds) per variant are shown as a box-and-whiskers plot with all individual data points indicated. (E to G) Data for the 3 nonpathogenic IL-6R variants are shown as described in the legends to panels B to D. All variants were compared to the IL-6R-WT with a one-way ANOVA with Dunnett’s multiple comparisons test. Statistically significant differences are indicated as follows: ****P < 0.0001.

These results suggest that the AF3 effect is not restricted to the IL-11R, but also applies to other proteins.

### Structural models of IL-6R and IL-11R variants with random point mutations are more similar to the known crystal structures than their WT models

So far, we have only analyzed naturally occurring variants of IL-6R and IL-11R. Therefore, we sought to further substantiate our findings by a complementary approach and generated 10 IL-6R variants with random mutations dispersed over the whole ectodomain (Fig. 4A). As shown in Fig. 4B to D, we observed the same effect as before, as all 10 random IL-6R variants matched the IL-6R crystal structure significantly better than the structural model of IL-6R-WT. In line with our previous results, 10 IL-11R variants with random mutations (Fig. 4E) performed significantly better than the IL-11R-WT model when compared to the IL-11R crystal structure (Fig. 4F to H). This suggests that the actual location of the point mutation has no influence on the observed effect.

**Fig. 4.**
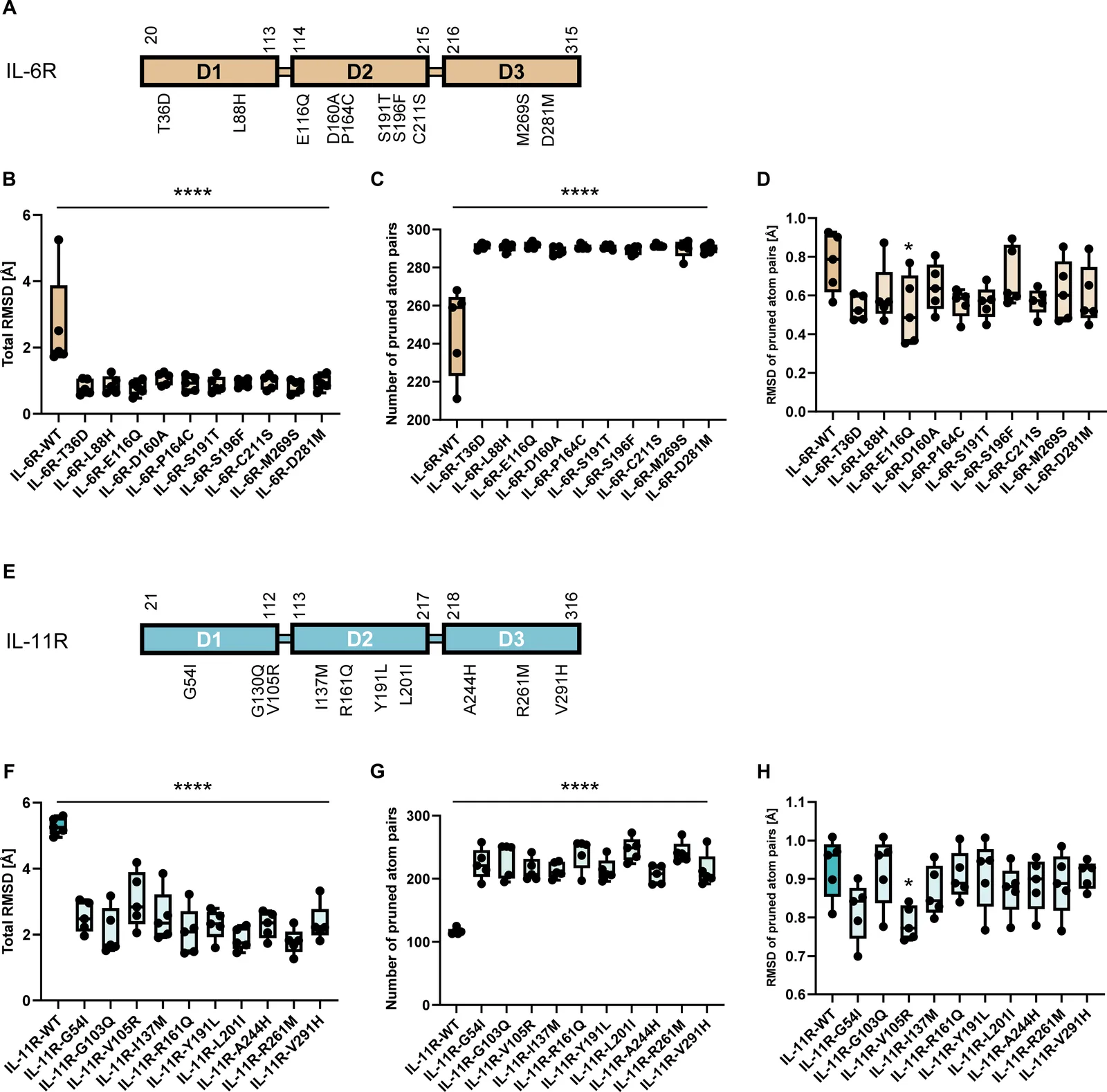
Structural models of IL-6R and IL-11R variants with random point mutations fit better to the known crystal structures than their WT models. (A) Schematic overview of the 3 extracellular domains (D1, D2, and D3) of the IL-6R. Numbers above the domains indicate the first and last residue according to [27]. The 10 IL-6R variants with random mutations are given below the IL-6R. (B to D) Total RMSD values (Å), the number of pruned atom pairs of the structural models, and the RMSD values (Å) of the pruned atom pairs are shown as described in the legends to Fig. 3. (E) Schematic overview of the 3 extracellular domains (D1, D2, and D3) of the IL-11R. Numbers above the domains indicate the first and last residue according to [27]. The 10 IL-11R variants with random mutations are given below the IL-11R. (F to H) Total RMSD values (Å), the number of pruned atom pairs of the structural models, and the RMSD values (Å) of the pruned atom pairs are shown as described in the legends to Fig. 1. All variants were compared to the IL-6R-WT or the IL-11R-WT, respectively, with a one-way ANOVA with Dunnett’s multiple comparisons test. Statistically significant differences are indicated as follows: *P < 0.05; ****P < 0.0001.

### Over-accordance of AF3-generated structural models with point mutations is caused by template bias

Finally, we hypothesized that the cause of the over-accordance of the structural models with point mutations compared to the WT might lie in the template usage. In the default settings, AF3 uses 4 templates for modeling a protein structure. Notably, AF3 used the same 4 templates (PDB: 6o4p, 1n26, 5fuc, and 7dc8) for all 22 naturally occurring IL-11R variants analyzed in this study (Fig. 5A). Interestingly, for IL-11R-WT, AF3 did not use the best-matching template 6o4p, but instead 3ew3 as the fourth template (Fig. 5A). We observed the same for the IL-6R: Prediction of all 5 variants was based on the same 4 templates (PDB: 1n26, 5fuc, 7dc8, and 1p9m; Fig. 5B), and again the best-matching template 1n26 was not used for the IL-6R-WT models. Here, 6o4p was used as the fourth template (Fig. 5B). This observation suggests that AF3 can select and rank different templates already for single-point mutants compared to the WT sequence, as AF3 can filter out templates with high sequence similarity to the input sequence [12].

**Fig. 5.**
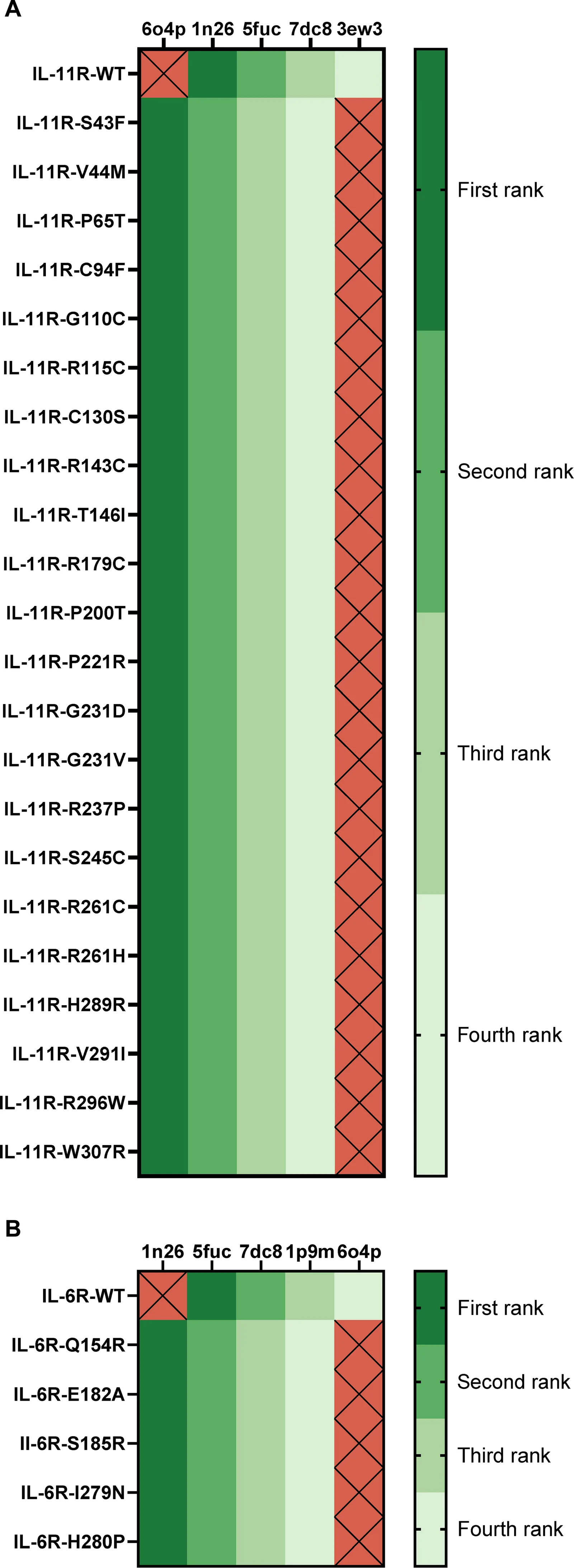
Over-accordance of AlphaFold 3 (AF3)-generated structural models with point mutations is caused by template bias. (A and B) Heat maps of the templates used by AF3 for the generation of the structural models of the (A) IL-11R and (B) IL-6R variants. Ranking of the templates from the highest rank to the lowest rank is indicated by the color code.

### No template usage removes over-accordance of AF3-generated structural models with point mutations

To confirm the hypothesis that usage of templates causes the over-accordance of structural models generated by AF3, we re-analyzed structural models of the IL-11R variants (Fig. 6A to C) and the IL-6R variants (Fig. 6D to F) with random mutations, modeled without the usage of templates. Indeed, modeling without templates did not show any measurable difference between the mutant and the WT models except for the number of pruned atom pairs for one of the IL-11R variants (Fig. 6A to F), identifying template bias as the cause for unexpected differences between models. In addition, when we forced AF3 to use a pre-determined set of templates for the IL-11R and IL-6R variants, again no significant differences between the WT receptors and their variants were determined (Fig. 6G to L).

**Fig. 6.**
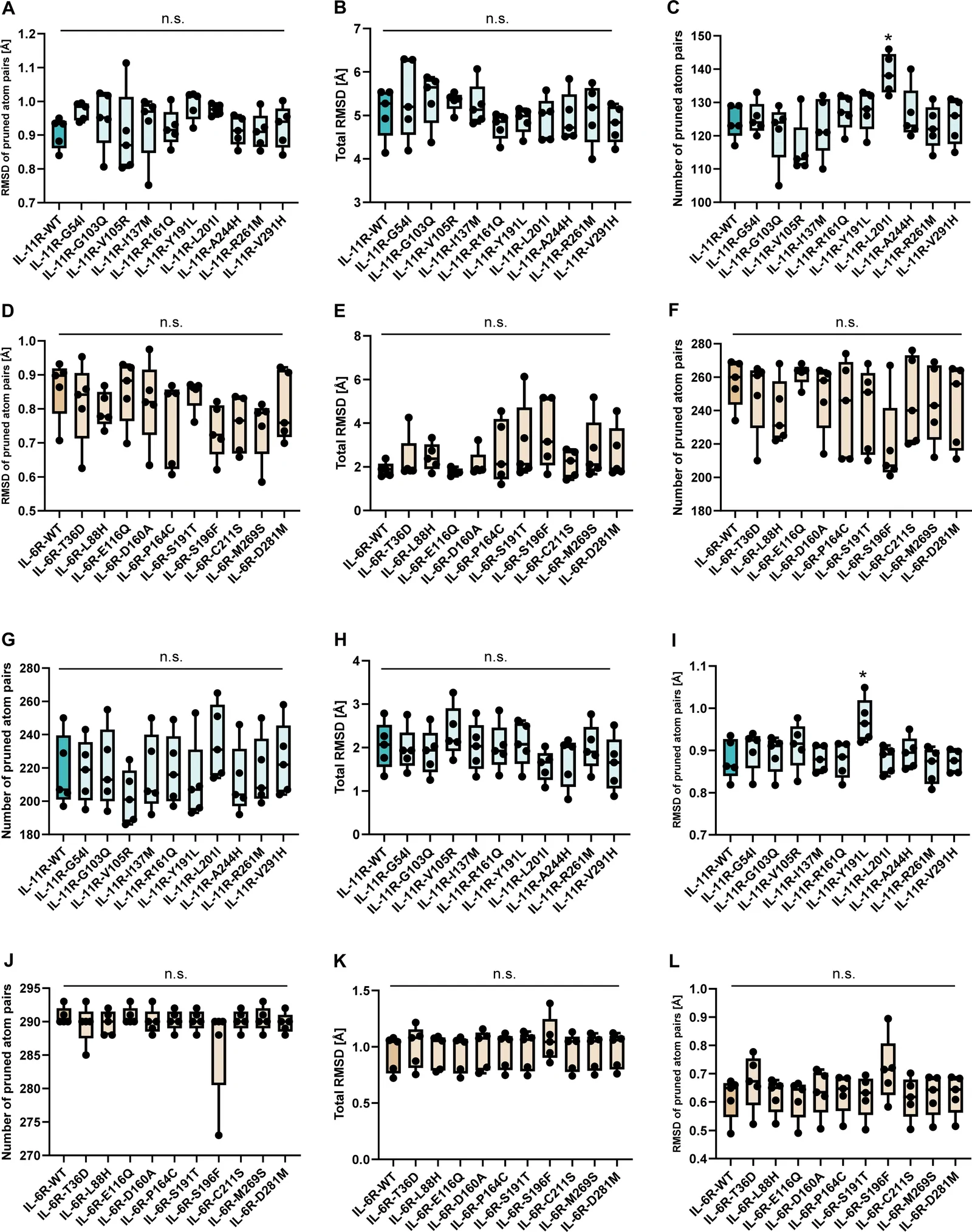
Over-accordance of AF3-generated structural models with point mutations is prevented by not using templates or by using a fixed set of templates. (A to C) Total RMSD values (Å), the number of pruned atom pairs of the structural models, and the RMSD values (Å) of the pruned atom pairs for the structural models generated without templates for IL-11R variants with random mutations are shown as described in the legend to Fig. 1. (D to F) Total RMSD values (Å), the number of pruned atom pairs of the structural models, and the RMSD values (Å) of the pruned atom pairs for the structural models generated without templates for IL-6R variants with random mutations are shown as described in the legend to Fig. 2. (G to L) The experiments were performed as described before, but with a fixed set of templates for all variants instead of using no templates at all. All variants were compared to the IL-6R-WT or the IL-11R-WT, respectively, with a one-way ANOVA with Dunnett’s multiple comparisons test. Statistically significant differences are indicated as follows: *P < 0.05; n.s., no significant difference.

### Analysis of IL-11R and IL-6R variants using AlphaMissense

Interestingly, when we analyzed the IL-11R and IL-6R variants using AlphaMissense, a deep learning model that builds on AlphaFold2 and was specifically developed to predict the effect of single amino-acid substitutions [31], we observed rather mixed results. Of the 15 pathogenic IL-11R variants, AlphaMissense classified only 9 as likely pathogenic, 4 as likely benign, and 2 as ambiguous (Fig. 7A). Of the 7 benign IL-11R variants, 5 were indeed classified as likely benign, but 2 were listed as ambiguous (Fig. 7B). For the IL-6R, as shown previously [32], one pathogenic variant was correctly classified, while the other variant was listed as being likely benign (Fig. 7C). Finally, 2 of the benign IL-6R variants were correctly determined, while the third one was ambiguous (Fig. 7D). These results show that AlphaMissense is able to correctly classify most, but not all, variants.

**Fig. 7.**
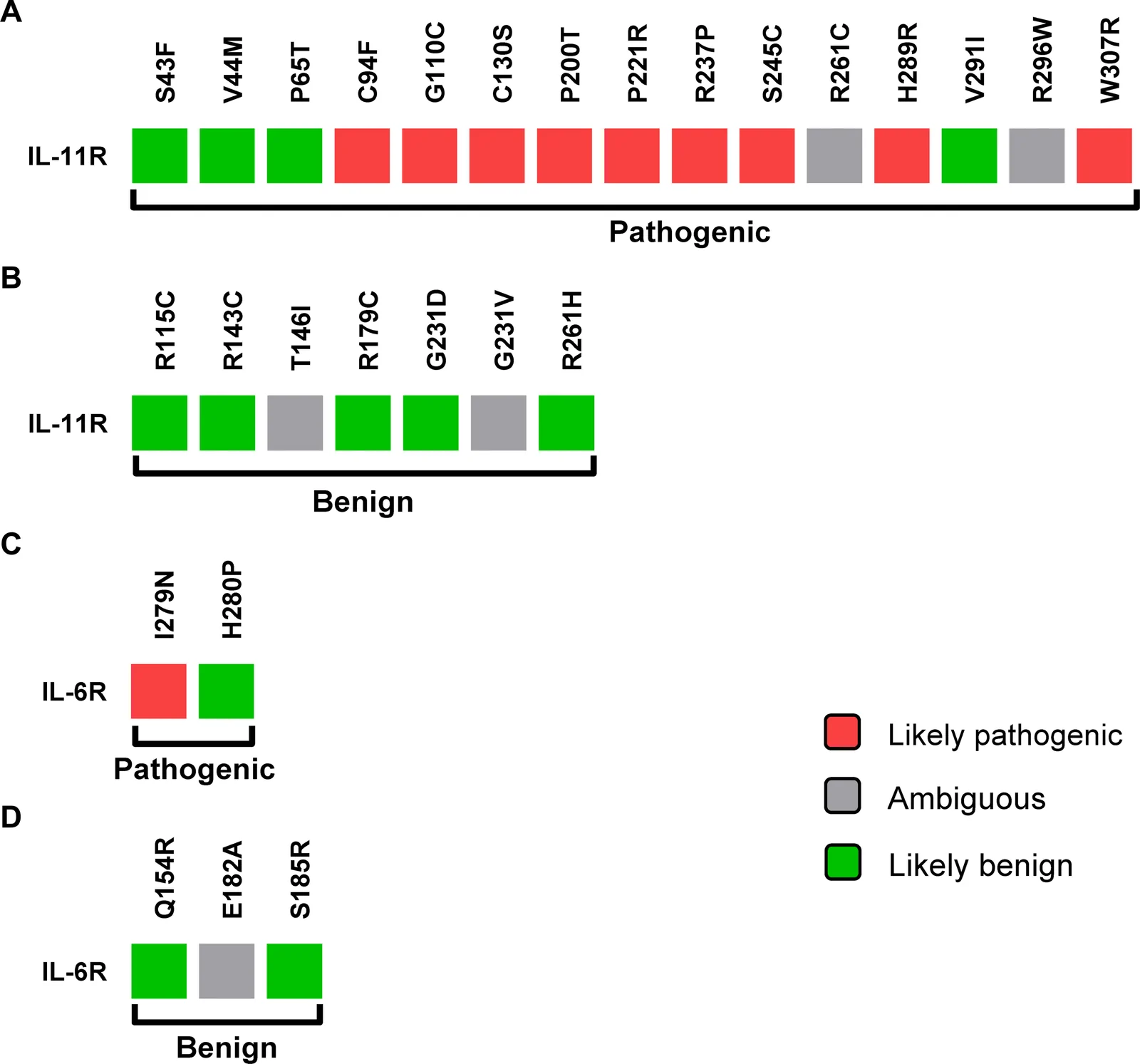
Analyzing IL-11R and IL-6R variants using AlphaMissense. (A to D) Pathogenic and benign variants of the (A and B) IL-11R and (C and D) IL-6R were analyzed using AlphaMissense. Variants classified as “likely pathogenic” are shown in red, variants classified as “likely benign” are shown in green, and variants classified as “ambiguous” are shown in gray.

## Conclusion

In summary, our results highlight that even single-point mutations can lead to different templates being identified and ranked in AF3. This means that WT and mutant predictions may not be based on the same underlying structural references. Therefore, the results obtained with AF3 in this regard have to be interpreted with great caution. A more appropriate strategy is to first predict the WT structure and subsequently introduce mutations for downstream analyses, such as side-chain relaxation, backbone relaxation, and molecular dynamics simulations, in order to evaluate mutation-induced changes in structure and stability. The recent development of AlphaMissense, which builds upon AF2, is another step toward using AI protein models for the analysis of point mutations and enabling their characterization in terms of being benign or pathogenic [31]. However, AlphaMissense was only able to correctly predict a fraction of the cytokine receptor variants in our analysis, highlighting that there is further need for improvement. Other approaches, e.g., combining AF3 predictions with mutagenesis tools like Jackal, have recently been demonstrated to offer additional molecular insights [33,34].

Our results are in agreement with a number of recent publications highlighting limitations of AF usage. Pratt *et al*. [35] reported that AF2, but not AF3, predicts confident but unrealistic structures for repeat proteins, and Chidambara Thanu et al. [36] showed that template-based modeling is superior compared to AF3-generated models for insect odorant receptors.

In summary, the combination of in silico prediction tools with wet-laboratory in vitro or in vivo experiments is still the gold standard to analyze the influence of a particular point mutation on the biological function of a protein.

## Data Availability

The authors declare that the data supporting the findings of this study are available within the manuscript and from the authors on request.
