# Supplementary material for: Template Bias in AlphaFold 3 Impedes Analysis of Point Mutations
Source: Comput Struct Biotechnol J. 2026 Nov 12;35(1):0240. doi: 10.34133/csbj.0240 (PMC13755562; doi:10.34133/csbj.0240)
Supplement: Supplementary file 1 [file csbj.0240.f1.docx]

**Figure S1: Alignment of AF3-generated structural models of IL-11R variants with the IL-11R crystal structure. (A)** Assembly of 25 structural models of IL-11R-WT (brown) were aligned with the crystal structure of the IL-11R ectodomain (pdb:6o4p). **(B)** Assembly of 25 structural models of three pathogenic IL-11R variants (IL-11R-C94F, IL-11R-P200T and IL-11R-R296W, all in red) were aligned with the crystal structure of the IL-11R ectodomain (pdb:6o4p). **(C)** Assembly of 25 structural models of three non-pathogenic IL-11R variants (IL-11R-R115C, IL-11R-P200T and IL-11R-R296W, all in red) were aligned with the crystal structure of the IL-11R ectodomain (pdb:6o4p).

**Figure S2: AlphaFold 3 confidence metrics of the structural models of the craniosynostosis-associated IL-11R variants. (A)** Mean predicted align error (PAE) matrices of IL-11R-WT and the different IL-11R variants are shown on the left. For each variant, the corresponding ΔPAE matrix, calculated as variant minus IL-11R-WT, is shown on the right. Dashed lines indicate the position of the respective amino-acid substitution. **(B)** Mean predicted local distance difference test (pLDDT) profiles of IL-11R-WT and the different IL-11R variants are shown. Dashed lines indicate the position of the respective amino-acid substitution. **(C)** Predicted template modeling (pTM) scores of all prediction models of IL-11R-WT and the different IL-11R variants are shown together with their respective means ± SD.

**Figure S3: AlphaFold 3 confidence metrics of the structural models of the IL-11R variants derived from gnomAD. (A)** Mean predicted align error (PAE) matrices of IL-11R-WT and the different IL-11R variants are shown on the left. For each variant, the corresponding ΔPAE matrix, calculated as variant minus IL-11R-WT, is shown on the right. Dashed lines indicate the position of the respective amino-acid substitution. **(B)** Mean predicted local distance difference test (pLDDT) profiles of IL-11R-WT and the different IL-11R variants are shown. Dashed lines indicate the position of the respective amino-acid substitution. **(C)** Predicted template modeling (pTM) scores of all prediction models of IL-11R-WT and the different IL-11R variants are shown together with their respective means ± SD.

**Figure S4: Alignment of AF3-generated structural models of IL-6R variants with the IL-6R crystal structure. (A)** Assembly of 25 structural models of IL-6R-WT (brown) were aligned with the crystal structure of the IL-6R ectodomain (pdb:1n26). **(B)** Assembly of 25 structural models of the two pathogenic IL-6R variants (IL-6R-I279N and IL-6R-H280P, both in red) were aligned with the crystal structure of the IL-6R ectodomain (pdb:1n26). **(C)** Assembly of 25 structural models of three non-pathogenic IL-6R variants (IL-6R-Q154R, IL-6R-E182A and IL-6R-S185R, all in red) were aligned with the crystal structure of the IL-11R ectodomain (pdb:1n26).

**Figure S5: AlphaFold 3 confidence metrics of the structural models of pathogenic and non-pathogenic IL-6R variants. (A)** Mean predicted align error (PAE) matrices of IL-6R-WT and the pathogenic IL-6R variants are shown on the left. For each variant, the corresponding ΔPAE matrix, calculated as variant minus IL-6R-WT, is shown on the right. Dashed lines indicate the position of the respective amino-acid substitution. **(B)** Mean predicted local distance difference test (pLDDT) profiles of IL-6R-WT and the pathogenic IL-6R variants are shown. Dashed lines indicate the position of the respective amino-acid substitution. **(C)** Predicted template modeling (pTM) scores of all prediction models of IL-6R-WT and the pathogenic IL-6R variants are shown together with their respective means ± SD. **(D-F)** Data for the non-pathogenic IL-6R variants are shown as described for panels A-C.
